# Denture Base Composites: Effect of Surface Modified Nano- and Micro-Particulates on Mechanical Properties of Polymethyl Methacrylate

**DOI:** 10.3390/ma13020307

**Published:** 2020-01-09

**Authors:** Touraj Nejatian, Neil Nathwani, Louise Taylor, Farshid Sefat

**Affiliations:** 1 Eastman Dental Institute, University College London, London WC1X 8WD, UK; tnejatian@yahoo.com (T.N.); n.nathwani@ucl.ac.uk (N.N.); 2 Department of Biomedical and Electronics Engineering, School of Engineering, University of Bradford, Bradford BD7 1DP, UK; l.woolley@bradford.ac.uk; 3 Interdisciplinary Research Centre in Polymer Science & Technology (IRC Polymer), University of Bradford, Bradford BD7 1DP, UK

**Keywords:** polymethyl methacrylate, flexural strength, impact strength, fracture toughness, filler particle, silane treatment

## Abstract

The most commonly used denture base material, polymethyl methacrylate, lacks ideal mechanical properties, which are reflected in its relatively high failure rate. Several methods have been explored to reinforce the material and reduce the cost of denture repair and replacement. In this study, various surface modified filler particles at different concentrations were dispersed in conventional and high-impact denture base materials and tested for their improvement in mechanical properties. Inorganic filler particles were coated with different silane coupling agents using an ultrasonic device. The particulates were dispersed in the resin and the composites polymerised through an innovative dual-cure technique. Charpy impact test, single-edge notch three-point bend fracture toughness test and Biaxial Flexural Strength (BFS) were performed on the specimens. The results showed that mechanical properties of the denture base resin can be improved by incorporating filler particles; however, the surface characteristics, quantity and level of dispersion of the particles play critical role in the mechanical behaviour of the composites. The results of this study are a promising step towards developing more fracture-resistant denture base materials.

## 1. Introduction

The most common used material for making dentures is polymethyl methacrylate (PMMA) owing to several advantages including accurate fits, stability within the oral environment, cost-effectiveness and simple laboratory and clinical manipulation [1]. Despite the common use of PMMA, two-thirds of dentures break within three years of their use [2,3]. Results from Darbar et al., 1994 [4], reported 33% of repairs were due to debonding of the teeth, 29% due to midline fractures and the remaining 38% due to other types of fracturing. The transverse strength of denture base materials is an indicator of performance [5,6] related to flexural fatigue and impact fracture [7].

Improvement of PMMA dentures would require four aspects to be considered, dependent on the etiology of the fracture: (a) maintaining mechanical characteristics through corrective surgery of anatomical abnormalities, to improve denture fit and balance the occlusion [8]; (b) optimizing chemical structure whilst forming the denture by modifying the packing and processing techniques [9,10]; (c) improving adhesion between teeth and denture base [11]; (d) modifying the composition chemically by replacing brittle polymers with adding rubber particles [12], adding fibres, metal inserts and other particles [13] polyamides, epoxy resin, PVA, polycarbonate, polyurethane and nylon [14,15,16].

**Figure 1:**
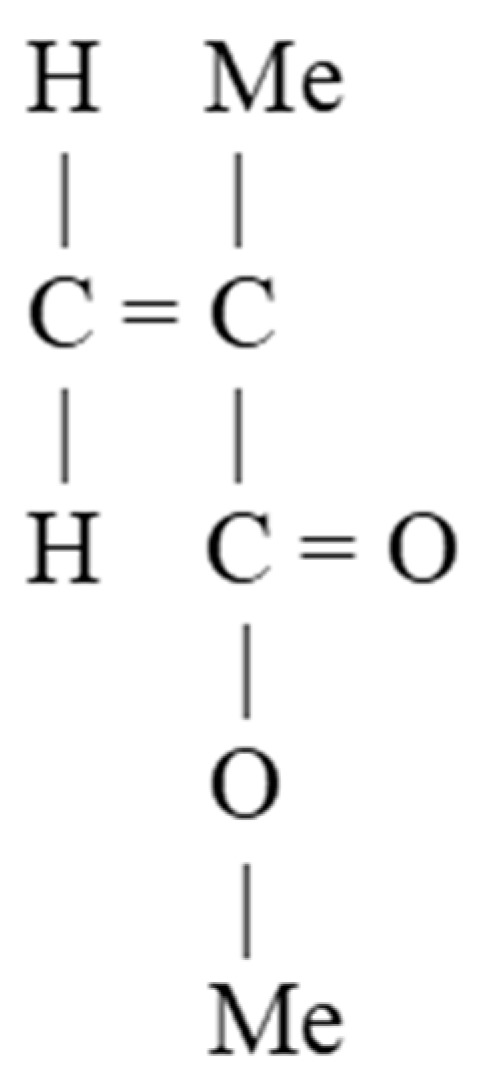
Methylmethacrylate molecular structure (Me stands for *-CH*3).

Conventional PMMA denture base material is mainly composed of pre-polymerised beads (PMMA powder) and monomer (Methyl Methacrylate, MMA). Methylmethacrylate monomers (Figure 1), using the process of free radical addition polymerization, make polymethylmethacrylate (PMMA). This process includes the sequence of activation, initiation, propagation and termination. Depending on the activation process, the materials are classified as heat-cured, cold-cured, light-activated or microwave-cured acrylic resin.

PMMA undergoes *addition polymerization* in which free-radical polymerization reactions occur with molecules containing carbon double bonds. This is a reaction without a by-product accelerated by heat, light or chemicals. The reaction is initiated by a free radical made by one of these initiators. The initiation stage is followed by the propagation stage in which other monomers bound to the free radical speedily shift the free electron to the end of the chain. The chain continues growing until free radicals are terminated. The termination stage can take place in several ways. Another material may react with a free radical can reduce the initiation or increase the rate of termination. The degree of monomer conversion depends on the method and condition of activation such as light activation versus dual activation, the distance from the light source and preheating prior to light activation. Decreasing the initiation rate retards polymerization and increasing the rate of termination reduces the degree of polymerization and the molecular weight of the final polymer. The polymerisation is inhibited by oxygen [17], hydroquinone and eugenol. Therefore, a small amount of hydroquinone is commonly presented in the monomer to increase the shelf life.

Conventional denture base materials are heat-cured and usually supplied as a powder and liquid. Composition of the powder included beads or granules of poly methyl methacrylate, benzoyl peroxide as an initiator, pigment/dyes, opacifiers-titanium/zinc oxides, plasticizer dibutyl phthalate and synthetic fibres-nylon/acrylic. Components of the liquid are methyl methacrylate monomer, hydroquinone and cross-linking agent-ethylene glycol dimethacrylate. Good physical properties are achieved by the high degree of polymerization, but the adaptation to the tissues is poor due to polymerization contraction, thermal contraction and the strain accompanying stress release during deflasking. Although heat-activated acrylic resins have certain flaws, they are still one of the most common used materials for denture fabrication.

Another type of heat-cure resin is rapid heat-polymerized which is a hybrid acrylic that is polymerized after 20 min in boiling water. The initiator combines both chemical and heat activated.

Inorganic filler particles have been used to reinforce polymers. Most of these particles are hydrophilic and have an affinity to agglomerate in a hydrophobic environment like PMMA therefore coupling agents are used to improve the fillers compatibility with organic matrices of the polymer. Tan et al. [18] and Chmielwska et al. [19] have developed separate silane treatment methods as particle surface modifiers, with 3-methacryloxypropyltrimethoxysilane (MPTS) being the most popular saline coupling agent. MPTS is bipolar molecule with hydrophilic and hydrophobic ends. It has the ability to bond to the inorganic filler particle through its hydrophilic end. The hydrophobic end of MPTS has alkoxysilane groups that bond to neighbouring MPTS molecules and copolymerised with acrylic monomers [20].

MPTS is absorbed into the particle surface by a hydrogen bond between the silanol group of MPTS and hydroxyl group of the particle surface. These hydrogen bonds are loose and are assumed to be converted into a covalent bond after drying at room temperature [21].

Previous attempts to improve properties of PMMA have resulted in improvement of some mechanical properties at the cost of other advantageous [22,23]. This is assumed to be due to poor compatibility and uneven distribution of the particulates within the polymer.

## 2. Aim

**Figure 2:**
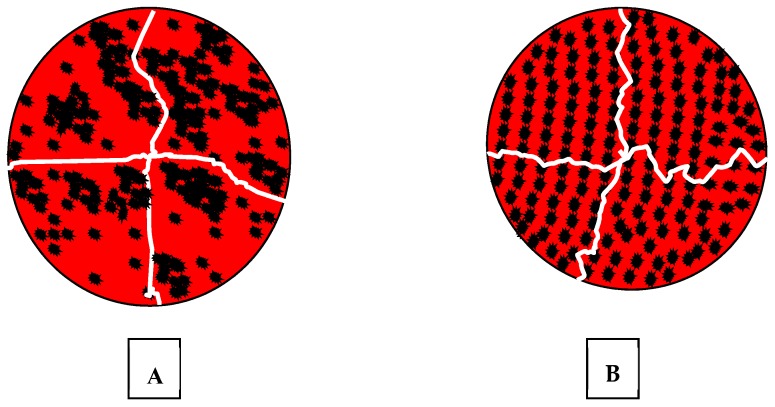
Schematic distribution of filler particles within the resin; (**A**) agglomerated, weekly bonded and randomly distributed particulates allow cracks to grow pass by them without obstruction. (**B**) uniformly dispersed particles, bonded to the resin matrix, divert cracks with increased fracture toughness of the material.

This study was designed to improve PMMA fracture resistance by modifying the surface of the particles and dispersing particles uniformly throughout the polymer. This experiment comprises of three stages: (I) Developing a technique to polymerise the monomer without mixing with PMMA powder to be able to disperse the particles within the whole polymer bulk; (II) modifying the surface of the filler particles to create a good bonding with resin matrix; (III) dispersion of the surface-modified particles within the monomer, preventing agglomeration (Figure 2).

## 3. Materials and Methods

### 3.1. Materials

TiO_2_ (Titanium oxide Anhydrous, Fisher Scientific UK Ltd., Bishop MeadowRoad, Loughborough, Leicestershire, UK) particles with a particle size of around 25 µm. ZrO_2_ (Zirconium Dioxide, Fisher Scientific UK Ltd., Bishop Meadow Road, Loughborough, Leicestershire, UK) with particle size of 25 µm. Al_2_O_3_ (Aluminium oxide, Fisher Scientific UK Ltd., Bishop Meadow Road, Loughborough, Leicestershire, UK) particles with a particle size of around 25 µm. PMMA—silica nanocomposite, 264 nm (prepared with 20 nm aqueous silica sol and surface characterised by Department of Chemistry of Sheffield University) Closite 30B, 20A, 93A and laponite (Southern Clay Products—a synthetic layered silicate incorporating an inorganic polyphosphate peptiser). Methyl methacrylate monomer (Oracryl denture material, Bracon, Etchingham, West Sussex, UK). Benzoyl peroxide 70% (Sigma-Aldrich Company Ltd., St. Louis, MO, USA). N,N dimethyl–p-toluidine (Sigma-Aldrich Company Ltd.) Silane-treated silica particles (Dentspy Ltd., York, PA, USA). Acrylic coated glass flakes (0.8–1.2 µm) (Glass Flakes Limited, Froster st., Leeds, UK). 3-methacryloxypropyltrimethoxysilane (MPTS) (Onichem Specialities Co., Ltd., Dalian, China). Enigma High-Base (Davis Schottlander & Davis LTD, Letchworth, UK).

### 3.2. Methods

#### 3.2.1. Stage I—Polymerisation of MMA

The authors found no studies in which the filler particles were distributed in the polymer beads and the monomer. The filler particles are mostly incorporated in monomer part of the material, which is almost one third of the polymerised form. This means that the particles distributed within the matrix only. In this study particles were dispersed in the monomer and polymerised without adding polymer beads. Therefore, a series of innovative procedures were tried to develop a technique for polymerisation of MMA without adding PMMA powder.

**Figure 3:**
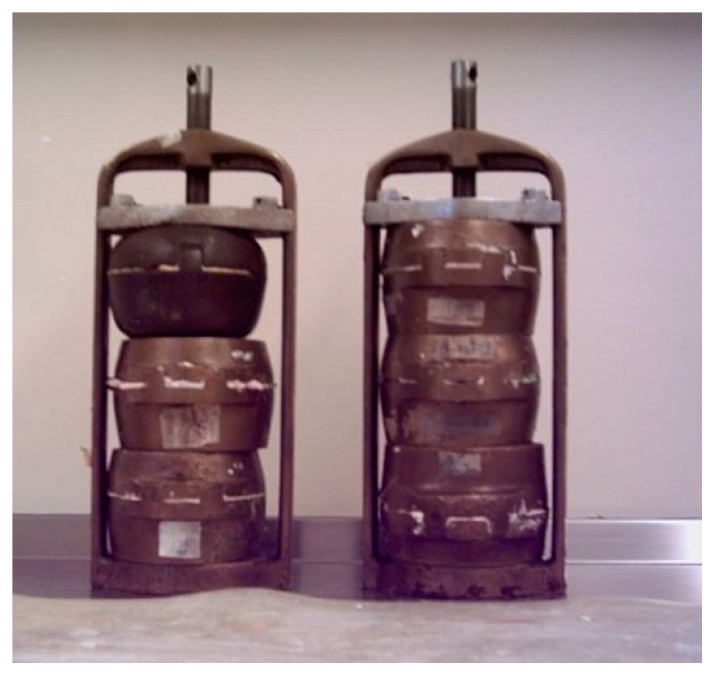
Conventional two-part brass flasks used in dental laboratories.

One hundred and eighty, grams (~200 cc) of monomer, MMA, was mixed with 1.8 g benzoyl peroxide 70% (1 w %), as a heat-cure initiator, then was poured in a plaster mould prepared in a two-part brass flask (Figure 3). Two sets of flasks were prepared and heated to 95 °C and 60 °C for 2 h in a dry-heat oven. This attempt at polymerisation failed due to monomer evaporation before polymerisation could take place. The experiment was repeated with a sealed mould, which resulted in malformed, porous and shrunken polymerised specimens from high volume of contraction on the monomer during polymerisation. An alternative method was designed where 1.8 g (~1 w %) of a chemical initiator, N,N dimethyl–p-toluidine, was added to the mixture of 180 g of MMA and 1.8 g of benzoyl peroxide and mixed with a magnetic stirrer for 50 min. This was poured into the mould as the mixture started to gel. The mould was stored for 24 h at ambient temperature for chemical curing then heated in a water bath at 95 °C to complete the polymerization. Visual inspection of the polymerised specimen showed that this dual-curing technique eliminated the problems of shrinkage and porosity of the resin. To reduce curing time to 10 min, the amount of benzoyl peroxide and chemical initiator were increased to 2 w % and 3 w %.

#### 3.2.2. Stage II—Surface Modification of Particles

To bond the inorganic filler particles to the resin matrix, the surface of the particles was impregnated with silane coupling agents. The authors could not find a commonly agreed procedure for silanation of various types of particles in the literature; therefore, they decided to develop a procedure using methods employed in previous studies and some innovative techniques as follows.

The control group was silane-treated silica particles dispersed using the same method of dispersion as experimental particles. Nanoclay filler particles, Closite 30B, Closite 20A, Closite 93A, and micron size filler particles, TiO_2_, ZrO_2_ (both particle sizes of 25 μm), Al_2_O_3_-PMMA (264 nm), were mixed individually with 1.8 g~2 mL MMA by ultrasonication at a frequency of 40 KHz for 10 min. These particles were then treated with the following experimental methods adapted from previous studies carried out by Albaladejo et al. [24] and Brentel et al. [25], to discover the optimal conditions.

Method I—Ethyl alcohol, 99% solution, was used as a solvent and is pH altered to 4.5–5.5 with acetic acid. A total of 0.25 g of MPTS, a silane coupling agent, was mixed with 5 mL of the solvent by magnetic stirrer, Bibby Scientific Model 8212 Magnetic Hot Plates stirrer, for 5 min at 500 rpm and stored at room temperature for 15 min to be hydrolysed to silanol (hydrolyses of alkoxy groups). A total of 5 g of all experimental particles was dispersed in 25 mL of solvent individually using an ultrasonic processor, at 40 KHz, for 5 min and then mixed with 5 mL of MPTS coupling agent for a further 5 min. The mixtures were stored at room temperature for 24 h to form initial hydrogen bonding between the particles and MPTS and washed three times by ethanol, 99%, using a centrifuge (1500 rpm for 15 min) to eliminate excess coupling agent. The excess coupling agent was assumed to encourage agglomeration of the filler particles. The precipitated particles were dried at room temperature for 24 h before fully drying in a furnace at 100 °C for 2 h to covert hydrogen bonds to covalent bonds. The silanols coordinate with metal hydroxyl groups on the inorganic surface to form an oxane bond with elimination of water.

Method II—Ethyl alcohol, 70% solution, was used as a solvent and its pH altered to 4.5–5.5 with acetic acid. A magnetic stirrer was used to mix 0.25 g of MPTS with 1 mL of solvent for 5 min at 500 rpm. The mixture stored at room temperature for 15 min to hydrolyse the silane to silanol. Five grams of closite 30B, closite 20A, laponite, TiO_2_, ZrO_2_ and Al_2_O_3_ were treated individually with 5% hydrogen peroxide for 2 h to encourage formation of hydroxyl groups and washed twice using 99% ethyl alcohol using a centrifuge at 2000 rpm for 10 min. These particles were then dispersed in the ethyl alcohol for 30 min by ultrasonication, at 40 KHz, then mixed with of 1 mL 5 w % silane coupling agent solution and stirred for one hour. The silane coupling agent solution was produced by mixing 0.25 g of the coupling agent and 1 mL ethyl alcohol 70%. These mixtures were stored at room temperature for 24 h to form the initial hydrogen bonds. All mixtures were washed with ethyl 99%, alcohol using a centrifuge at 2000 rpm twice for 10 min. The precipitated particles were dried at room temperature for 24 h and then placed into a furnace at 100 °C for 2 h.

Method III—Following methods I and II, Al_2_O_3_ showed poor results; therefore, an alternative method was used to treat Al_2_O_3_ alongside TiO_2,_ ZrO_2_ to provide a comparison from similar micron-size particles. The solvent in this case was 70% Ethyl alcohol with pH adjusted to 4.5–5.5 by acetic acid. MPTS as silane coupling agent was added to the solvent with ratio of 0.25 mg/mL and stirred with a magnetic stirrer for 5 min. The mixture was then stored at room temperature for 15 min in order to hydrolyse silane to silanol. Five grams of TiO_2,_ ZrO_2_ and Al_2_O_3_ were individually treated with 5% hydrogen peroxide for 2 h and washed twice with distilled water using a centrifuge at 2000 rpm for 10 min. The particles were then dispersed in 95% ethyl alcohol for 30 min by ultrasonication, at 40 KHz, and mixed with 1 mL of 5 w % silane coupling agent solution. This produced 0.25 g of MPTS and 1 mL of 98% ethyl alcohol. This was stirred for an hour and then stored at room temperature for 24 h to allow the particles to become wetted by the coupling agent. The mixtures were centrifuged at 2000 rpm for 10 min, precipitated particles were dried at room temperature for 24 h, then placed into a furnace at 100 °C for 2 h.

**Figure 4:**
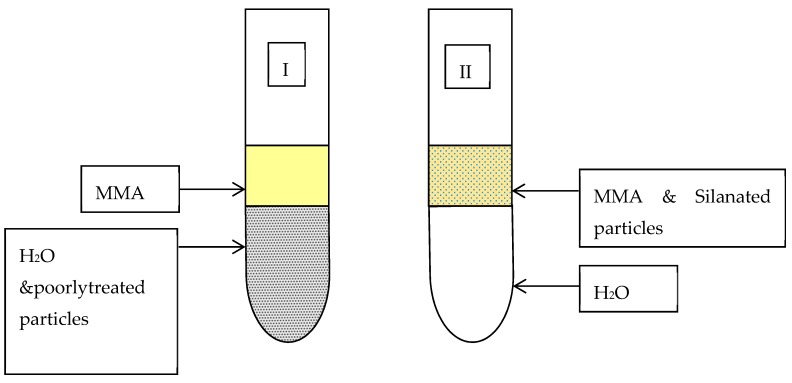
Qualitative method to assess the compatibility of silanated particles with MMA.

In all methods, a qualitative tube test (Figure 4) was used to assess the compatibilities of the particles before and after coupling treatment [26]. The surface-modified particles were mixed with monomer and added to a test tube, which was half filled with water. Well coated particles remained suspended in monomer whereas poorly treated particles settled in the water. All treated particles were compared to untreated particles after being stored for 1 and 24 h. The control group was commercial silane-coated silica glass particles.

#### 3.2.3. Stage III—Dispersion of Filler Particles within the Resin

To achieve uniform dispersion, particles were added into the MMA using an ultrasonic processor for 20 min, at 40 KHz, to break down the agglomeration. As an initiator, 2 w % of 70% Benzoyl peroxide along with 3 w % N,N-Dimethylptoluidine, as a chemical activator, were added to the mixture and stirred for 10 min. When the mixture started to form into a gel, it was transferred into the mould, packed and stored at room temperature for 10 min. The chemically curing resin was transferred into a water bath and cured at 95 °C for 2 h to complete the polymerisation. The moulds were either discs of 12 mm diameter and 2 mm thickness for the BFS test or bar-shaped 4 × 10 × 80 mm/4 × 4 × 50 mm for Charpy impact and single notch three-point bend fracture toughness tests, respectively. Single-edge notched specimens were notched in the middle using a diamond wheel (5 × 0.015 × 1/2 inch) to a depth of 2 mm. The ratio of notch depth/specimen width was kept in the range 0.45 to 0.55. Plain MMA was processed under the same condition as a control. After processing, the flasks were left to cool to room temperature, and specimens were removed with excess resin “flash” trimmed using a hand piece and a tungsten carbide bur. The specimens were further sandpapered with 600 μm silicon carbide to achieve completely flat surface and uniform thickness. The finished specimens were stored at 37 °C for 48 ± 2 h in tap water.

### 3.3. Control Materials

A high-impact resin, Enigma High-Base, was prepared according to manufacturer’s recommendations of 20 mg powder and 10 mL monomer to compare the improvements in mechanical properties of the resin. This was cured in a water bath at 95 °C for 2 h to form discs and bars for the BFS (N = 20), the impact strength (N = 10) and the fracture toughness (N = 10) tests. In addition, 1 w % of acrylic-coated glass flakes were mixed with Enigma high-base monomer by ultra-sonication for 10 min, at 40 KHz, and then mixed with the powder, packed and processed under the same conditions. A commercial plain acrylic denture base, Oracryl, was also prepared with a powder to monomer ratio of 2.5:1 and cured in a water bath at 95 °C for 2 h. All specimens were stored in a water bath at 37 °C for two days before testing.

### 3.4. Testing Method

**Figure 5:**
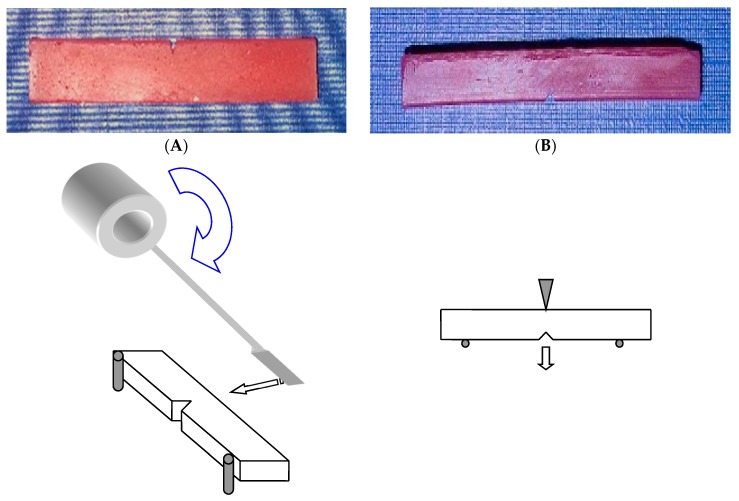
(**A**) Charpy impact test and specimen, (**B**) single-edge notch for fracture toughness test and specimen.

The Charpy specimens were loaded in an impact tester Model IT503 Plastic Impact Tester, Surrey, UK) with a span length of 76 mm (Figure 5A). The machine was calibrated before conducting the test. The impact test was carried out at room temperature, and the impact strength of the specimen was calculated automatically by the machine based on the energy absorption and the specimen geometry. The fracture toughness and BFS tests were performed using the tensile-testing machine at the cross speed of 0.5 and 1 mm/min, respectively. The fracture toughness specimens were loaded in the machine with a span length of 38 mm and centred notch using manufacturer jig (Figure 5B).

The following equation (Equation (1)) was used to calculate the fracture toughness of the specimens.

Equation (1): [27] where *P* = Peak Load, *S* = Span, *B* = Specimen Thickness, *W* = Specimen Width and *a* = Crack Length.

*KIc* = [(*P* × *S*)/(*B* × *W*^1.5^)]·ƒ (*α*/*w*) ƒ (*α*/*w*) = 3 (*α*/*w*) ^0.5^ [ 1.99 − (*α*/*w*) (1 − *α*/*w*) (2.15 − 3.93 *α*/*w* + 2.7 *α*^2^/*w*^2^)] ÷ 2 (1 + 2 *α*/*w*) (1 − *α*/*w*)^1.5^ (1)

A Lloyd 2000R universal testing machine, was used to measure BFS of the discs. The discs were centrally placed onto an “O” ring, and a ball tip instrument was used to apply load on the central point of the discs at a crosshead speed of 1 mm/min. The BFS of the specimens was calculated using the following equation (Equation (2)) in an Excel data file.

Equation (2): [28] where *σ_max_* = maximum biaxial flexural strength, *P* = the load to fracture, *α* = the radius of the knife-edge support (O ring) and *h* = specimen thickness

(2) σmax=ph2[0.606 loge(aw)+1.13]

The electron microscopic study was carried out on the fractured surfaces of plain resin, silanated silica-resin (2 w %), silanated TiO_2_–resin (1 w %) and silanated nanoclays-resin composites. The specimens were mounted on 12.5 mm diameter stubs and attached with sticky tabs and then coated in an Edwards S150B sputter coater with approximately 25 nm of gold. The specimens were then examined in a Philips XL-20 Scanning Electron Microscope at an accelerating voltage of 20 Kv.

### 3.5. Statistical Analysis

One-way ANOVA analysis and Turkey’s comparison tests were carried out to determine significance using MINITAB (*p* < 0.05).

## 4. Results

### 4.1. Surface Modification of Particles

**Figure 6:**
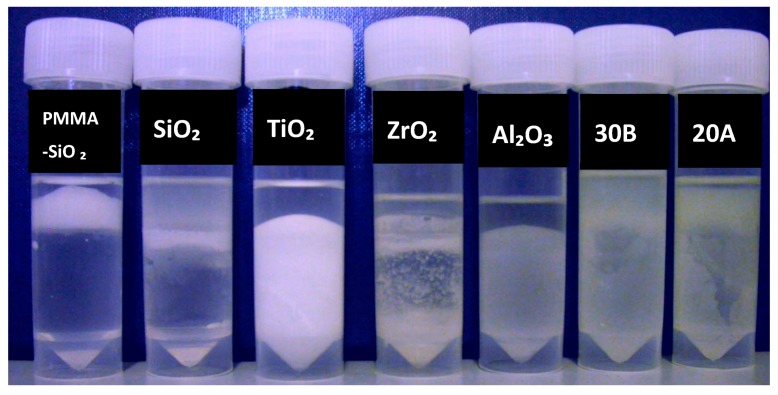
Particles stored for one hour after mixing with monomer, prior to silane treatment, from left to right; impregnated PMMA by silica, silane treated silica (control), untreated TiO_2_, ZrO_2_, Al_2_O_3_ and nanoclays 30B and 20A.

**Figure 7:**
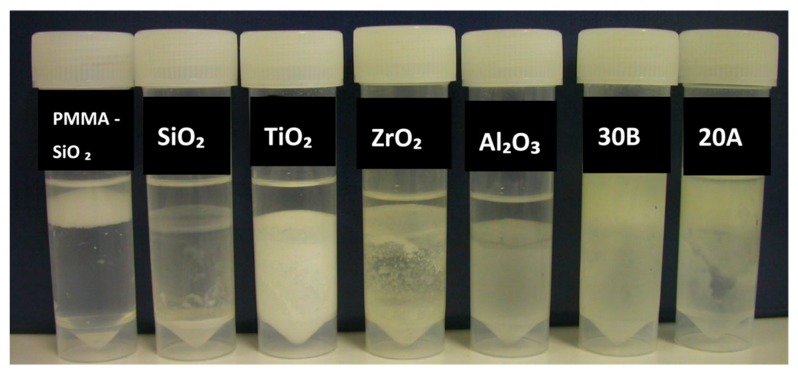
Particles stored for 24 h after mixing with monomer, prior to silane treatment, from left to right; impregnated PMMA by silica, silane treated silica, untreated TiO_2_, ZrO_2_, Al_2_O_3_ and nanoclays 30B and 20A.

After 1 h untreated TiO_2_ and Al_2_O_3_ were completely deposited in the water whereas ZrO_2_ partially did. Nanoclays, closite 20A and 30B were mostly suspended in the monomer, whereas silane-treated silica (control group) and PMMA-impregnated colloidal silica particles were almost completely suspended in MMA (Figure 6). After 24 h most of the silane-treated silica particles precipitated, while there was no visually significant change in the other specimens (Figure 7).

**Figure 8:**
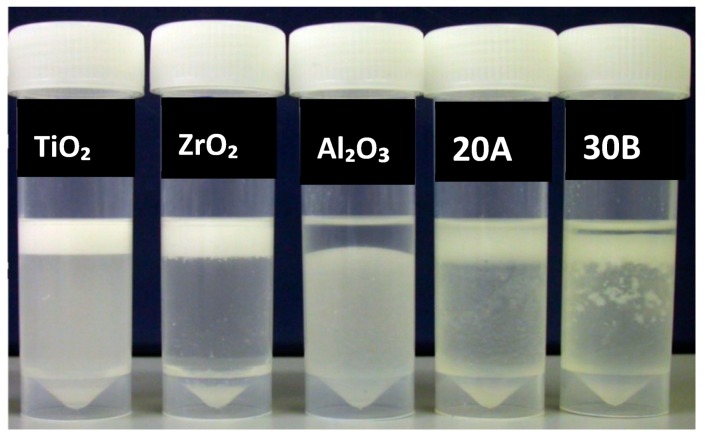
Particles subsequent to silane treatment, method I, mixed with monomer stored for one hour from left to right; silane treated TiO_2_, ZrO_2_, Al_2_O_3_ and closite 30B and 20A.

**Figure 9:**
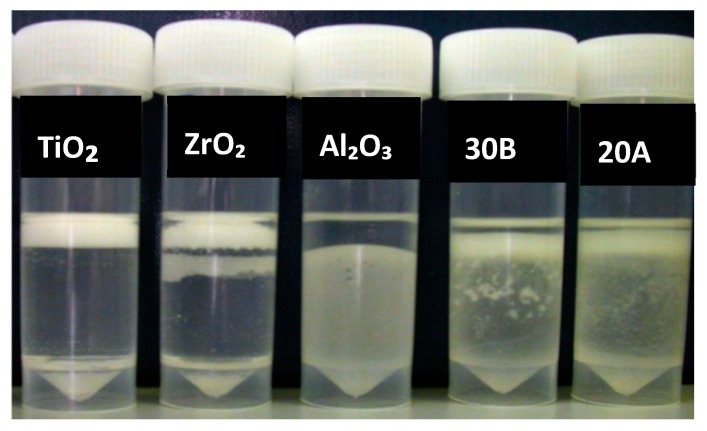
Particles subsequent to silane treatment, method I, mixed with monomer stored for 24 h from left to right; silane treated TiO_2_, ZrO_2_, Al_2_O_3_ and nanoclays 30B and 20A.

Silane-treated particles using the method I showed that after one hour, ZrO_2_ and TiO_2_ became almost completely suspended into the monomer, but the suspension of Al_2_O_3_, closite 20A and closite 30B had not changed (Figure 8). Further storage of the samples to 1, 3 and 7 days had no effect on the compatibility of the particles (Figure 9).

**Figure 10:**
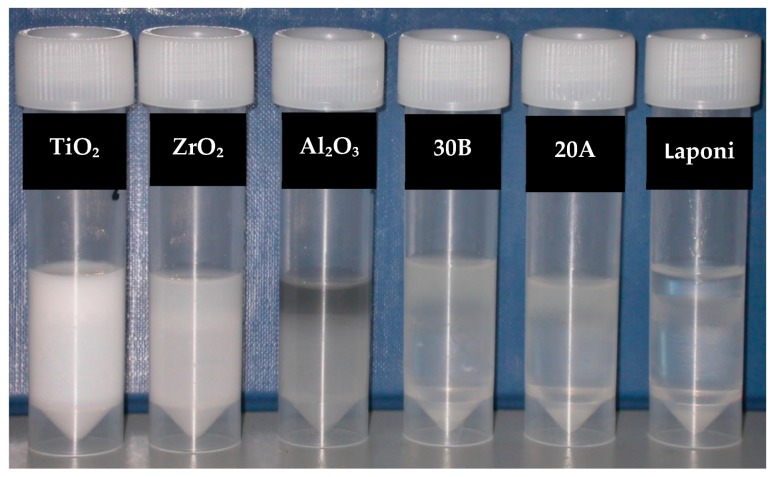
Particles subsequent to silane treatment, method II, mixed with monomer and stored for one hour from left to right; silane treated TiO_2_, ZrO_2_, Al_2_O_3_, Closite 30B, Closite 20A and laponite.

**Figure 11:**
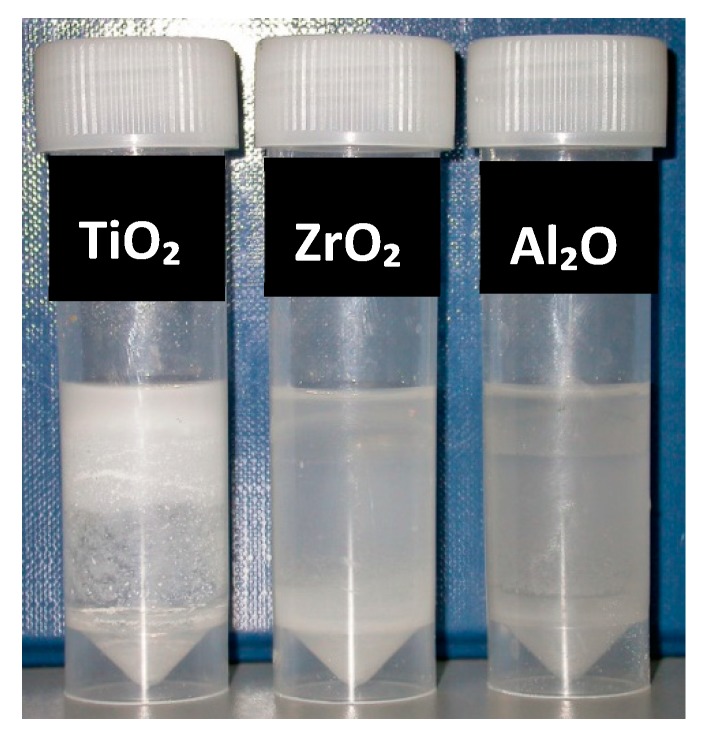
Particles subsequent to silane treatment, method III, mixed with monomer and stored for one hour from left to right; silane treated TiO_2_, ZrO_2_ and Al_2_O_3_.

The tube test of Silane-treated particles through method “II” showed that after 1-h storage, TiO_2_, ZrO_2_, Al_2_O_3_, closite 20A and closite 30B were partially suspended in the monomer. Silanated laponite was particularly compatible with MMA (Figure 10). Method III of silane treatment showed a medium degree of compatibility with the monomer after treatment (Figure 11).

### 4.2. Biaxial Flexural Strength of Adapted Resins

**Figure 12:**
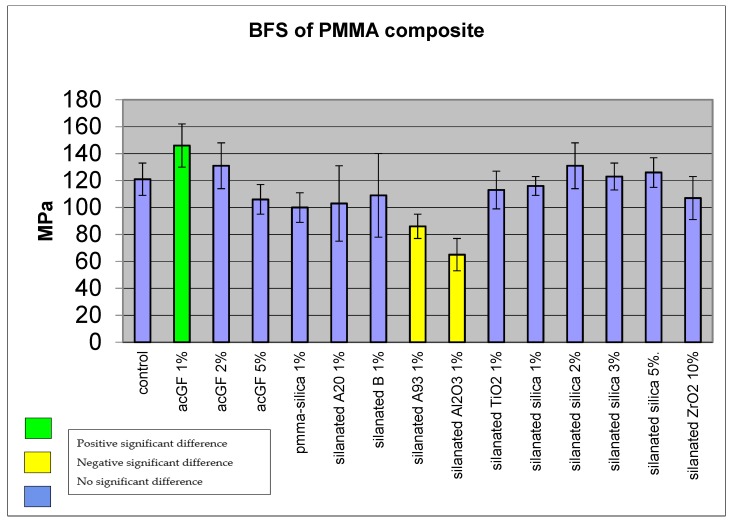
Comparison of biaxial flexural strength of filler added resin and plain resin.

Adding 1 w % of acrylic coated glass flakes improved the BFS of the resin to 146 MPa, SD ± 16 (N = 15) compared with the control group (polymerized plain monomer) which showed 121 MPa, SD ± 12. The difference was statistically significant (*p* < 0.05); however, increasing the particle ratio to 2 w % showed less improvement at 131 MPa, SD ± 17, which was not statistically significant (*p* > 0.05). Adding 5 w % of particles reduced the BFS of the resin to 106 MPa, SD ± 11, although the difference was not statistically significant (*p* > 0.05) (Figure 12 and Table 1). Adding PMMA-silica nanocomposite, silanated A20, B nanoclays, silanated TiO_2_ and ZrO_2_ reduced the mean BFS of the resin to 100, SD ± 11, 103, SD ± 28, 109, SD ± 31, 113, SD ± 14, 107, SD ± 16 MPa, respectively. However, the differences were not statistically significant (*p* > 0.05). Incorporation of silanated closite A93 1 w % and silanated Al_2_O_3_ 1 w % reduced the mean BFS of the resin to 86, SD ± 9 and 65, SD ± 12 MPa. The differences were statistically significant (*p* < 0.05). The mean BFS of 1 w % silanated silica composite was 116 MPa, SD ± 6. In comparison with the control group, the difference was not statistically significant (*p* > 0.05). Incorporation of 2, 3 and 5 w % of silanated silica into the resin increased the mean BFS of the resin to 131, SD ± 17, 123, SD ± 10, 126, SD ± 11 MPa. The differences were not statistically significant compared to the control group (*p* > 0.05).

### 4.3. Impact Strength of Resins

**Figure 13:**
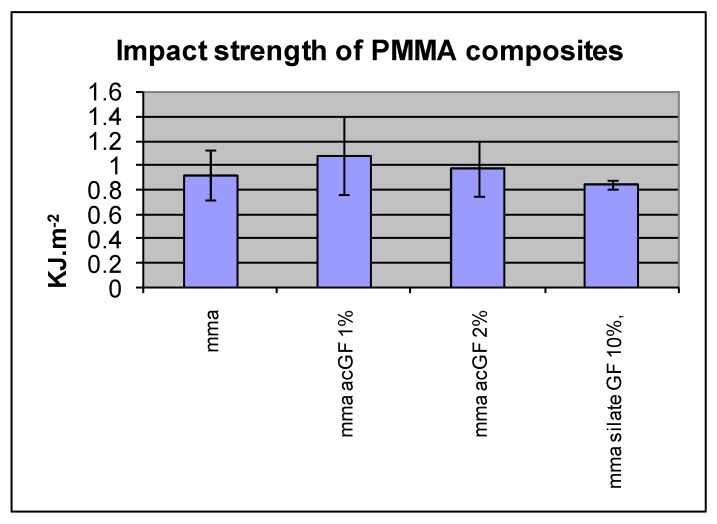
Effect of adding filler particles on impact strength of resins.

Incorporation of 1, 2 and 3 w % acrylic coated glass flakes into the resin did not improve the mean impact strength compared with the control group (N = 10). The differences were not statistically significant in any of these ratios (*p* < 0.05) (Figure 13 and Table 2).

### 4.4. Fracture Toughness of the Adapted Resins

**Figure 14:**
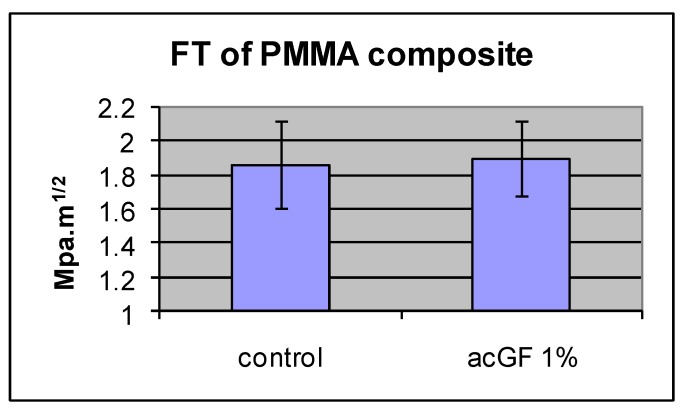
Effect of adding filler particles on fracture toughness of the resin.

Adding 1 w % of acrylic coated glass flakes to the resin did not improve the mean fracture toughness in comparison with the control group (N = 10). The difference was not statistically significant (*p* < 0.05) (Figure 14 and Table 3).

**Figure 15:**
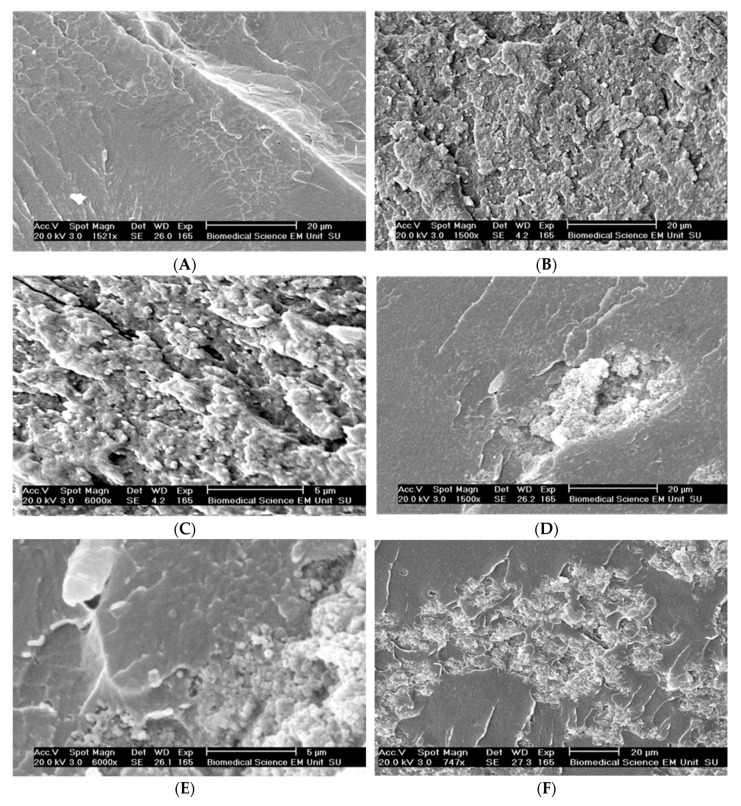

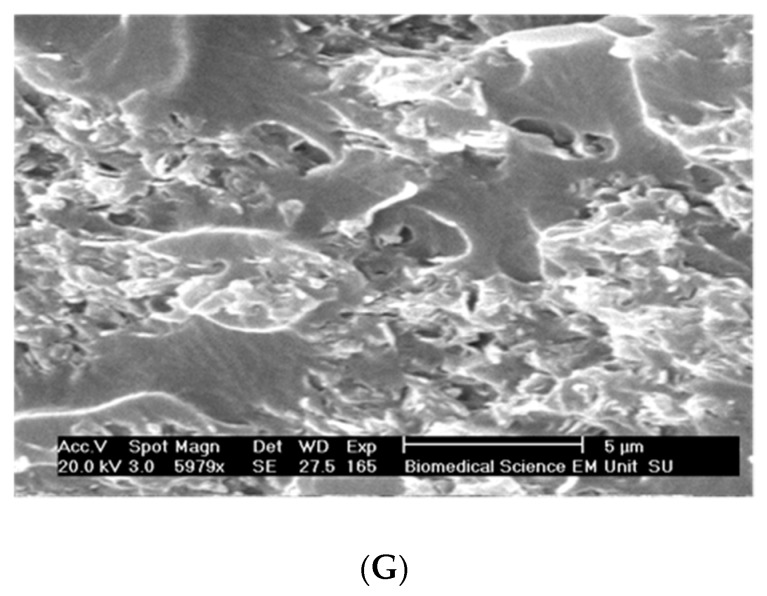
SEM images of fractured surfaces of the resins. (**A**) Plain resin. (**B**) Silanated silica-resin (2 w %). (**C**) Silanated silica-resin (2 w %). (**D**) Silanated TiO_2_-resin (1 w %). (**E**) Silanated TiO_2_-resin (1 w %). (**F**) Silanated nanoclay-resin composites. (**G**) Silanated nanoclay-resin composites.

To study the pattern and surface specification of the fractured surface, a Scanning Electron Microscope, SEM, was used. In SEM images, the rougher fracture surface of the silica composite indicates its higher energy absorption to fracture compared with the plain resin, and the particles seem to be dispersed uniformly. The scanning showed that silane treated TiO_2_ and nanoclay particles were still agglomerated in the resin, and the crack went through the clusters as weak spots (Figure 15).

### 4.5. High-Impact Resin Results

**Figure 16:**
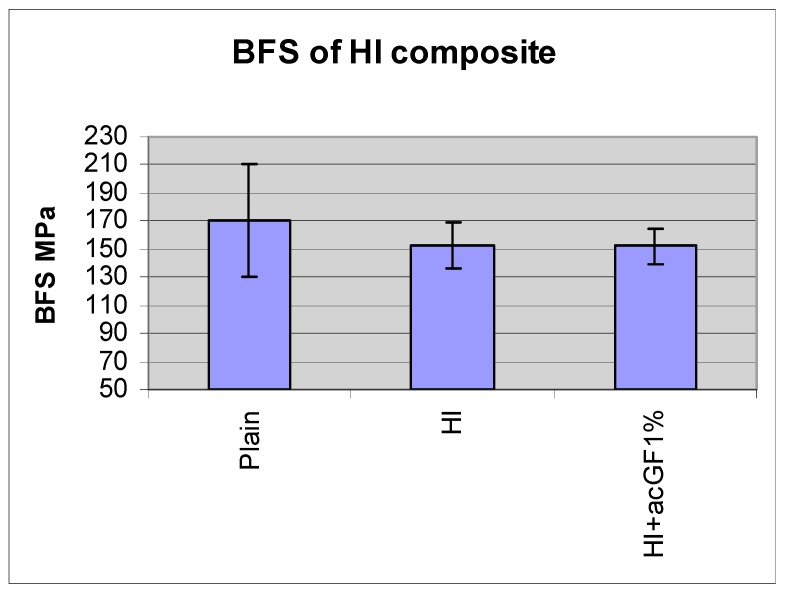
BFS of high-impact resin with and without added glass flakes, compared to plain denture resin.

The mean BFS of the high-impact resin and glass flake added (1 w %) high-impact resin were 153 MPa, SD ± 16 and 152 MPa, SD ± 13, respectively (N = 20). There was no statistically significant difference between them. The mean BFS of high-impact resin with or without the particles was not different than that of plain denture base resin 170 MPa, SD ± 40. The difference was not statistically significant (*p* < 0.05) (Figure 16 and Table 4).

### 4.6. Impact Strength Results

**Figure 17:**
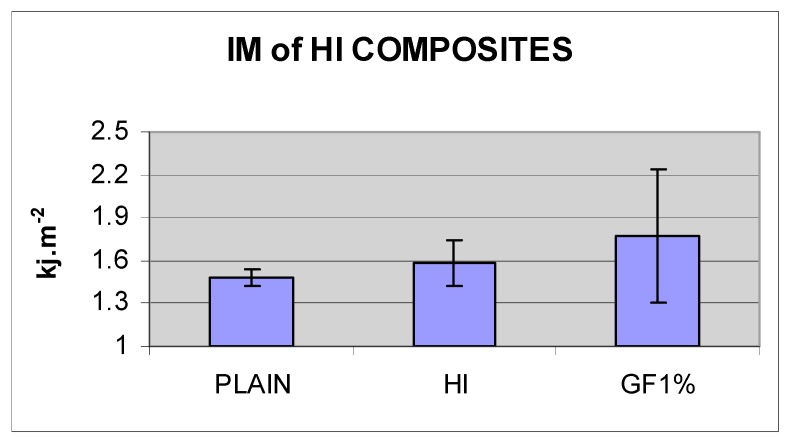
Impact strength of plain acrylic denture base resin, high-impact denture base resin and its composite with acrylic coated glass flakes.

The mean impact strength of plain and high-impact denture base resins was 1.48, SD ± 0.06 and 1.58, SD ± 0.16 KJ·m^−2^, respectively (N = 9). The difference between them was not statistically significant (*p* < 0.05). The incorporation of 1 w % acrylic coated glass flakes into the high-impact resin did not improve its mean impact strength. The difference between this composite and those of the two denture base resins was not statistically significant (*p* < 0.05) (Figure 17 and Table 5).

### 4.7. Fracture Toughness Results

**Figure 18:**
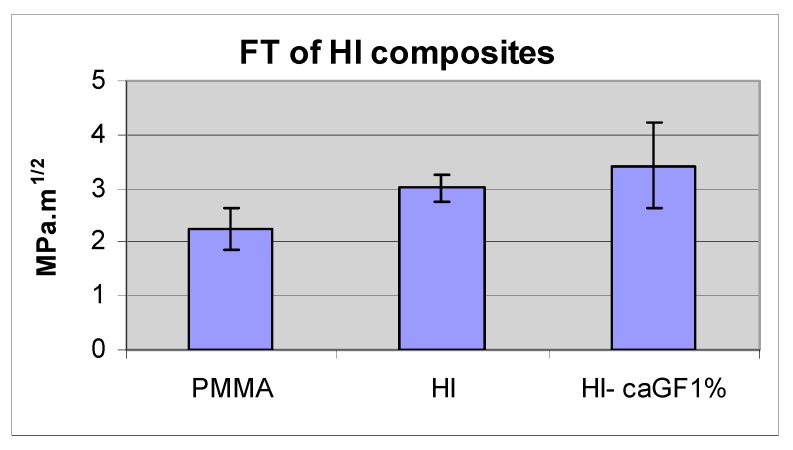
Fracture toughness of the plain acrylic denture base resin, high-impact denture base resin and its composite with acrylic coated class flakes.

The fracture toughness of plain and high-impact denture base resin was 2.24, SD ± 0.38 and 3.02, SD ± 0.25 MPa·m^1/2^, respectively. The difference between them was statistically significant (*p* > 0.05). Adding 1 w % of acrylic coated glass flakes to high-impact resin did improve its fracture toughness to 3.43 MPa·m^1/2^, SD ± 0.78. The difference between this composite and the high-impact resin was not statistically significant (N = 9) (*p* < 0.05) (Figure 18 and Table 6).

## 5. Discussion

### 5.1. Surface Treatment

The tube test of the particles revealed that the storage time of untreated inorganic micro and nanoparticles within the monomer did not affect their compatibility. The commercial silane-treated silica glass particles retained their compatibility with monomer for one hour; however, in the longer period, 24 h, they lost this property, which may be due to hydrolysis of oxane bond (Si-O-Si) between the coupling agent and the particle surface in an aqueous environment. The silane treatment of ZrO_2_ and TiO_2_ particles improved their compatibility with MMA, which was stable at least for one week; however, silanation of nanoclays had no or limited effect on their compatibility with the hydrophobic environment. The results showed that compatibility of Al_2_O_3_, ZrO_2_ and TiO_2_ could be altered by implementing different silanation techniques. This finding is consistent with results of previous studies [20]. Hydrogen peroxide pre-treatment prior to coupling agent treatment in Methods II and III was employed to increase the number of the hydroxyl group on the particle surface and seems to be effective in the case of Al_2_O_3_. However, this technique reduced the compatibility of ZrO_2_ and TiO_2_ and had no effect on nanoclays in comparison with the results of method I. This difference is assumed to be created by the different physical and chemical characteristics of the particles. The test tube results also showed that rinsing the TiO_2_ and Al_2_O_3_ particles after coupling agent treatment in method II decreased their compatibility with the resin in comparison with unwashed particles in method III. This is consistent with the result of a study conducted by Liu et al. [21] in which loosely adsorbed coupling agent was washed out due to lose bonding to the particle surface. Therefore, the silane treatment technique should be optimized for each individual type of the particles.

### 5.2. Mechanical Properties of Filled Conventional Acrylic Resin

**Figure 19:**
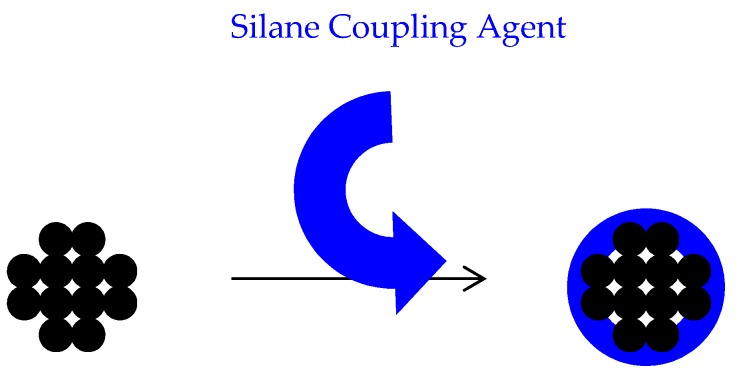
Speculated graphic feature of a coupling agent covered particle cluster.

The incorporation of 1 w % acrylic coated glass flakes improved the BFS of the resin, which may be because of their uniform dispersion and physical or chemical bonding to the resin matrix due to reducing inherent flaw size. This enables efficient stress transfer between the filler particles and the resin matrix. However, increasing the ratio of the particles to 5% decreased the strength compared with that for 1 w %. This result is in accordance with the finding of other studies by Adabo et al. [29] and Mortazavi et al. [30]. A possible explanation for this is the fact that the more particles are introduced to the resin, the more likelihood of agglomeration will occur within the resin. The clustered particles can potentially act as a void in the resin and therefore reduce the overall strength of the resin. Despite showing good compatibility with monomer, TiO_2_ and ZrO_2_ had no significant effect on the BFS of the resin possibly because of agglomeration of the particle prior to silane treatment that resulted in silane-coated clusters. These clusters, although suspended in the monomer, may act like a void in the cured resin (Figure 19). This is further supported by SEM images taken from the fracture surface (Figure 15D,E). In addition, the spatial orientation of the silane molecules and the creation of cross-linked multilayer coupling agent around the particles reduced the number of hydrophobic functional groups available to react with matrix monomer [20,31]. This result is consistent with the finding of a similar study in which silanated zirconium oxide nanoparticulates were incorporated into the matrix of PMMA [32]. However, another study showed incorporation of ZrO_2_ nanoparticles improved the bending strength and fracture toughness of high-impact denture materials at the cost of impact strength [23]. The difference is probably due to the different particle size and testing methods used in this experiment.

The addition of PMMA-silica nanocomposite (PMMA grafted silica particles) into the resin, despite good compatibility with monomer, did not improve the strength of the resin. This may be because of a highly cross-linked PMMA shell around the silica particles, which did not react chemically with the matrix resin. Silanated Closite 20A and 30B nanoclays did not improve the BFS of the resin. This finding matches their poor compatibility with monomer and intercalated structure, which can be exfoliated when they are subjected to stress. This is consistent with the finding of Fu and Naguib [33], who revealed the agglomeration of nanoclays in acrylic resin and its negative effect on the mechanical properties of the resin. This is in contrast with findings of another research in which adding 0.25 and 0.5 wt % of double-modified nanoclay improved flexural strength, flexural modulus and fracture toughness of acrylic denture base [34]. This can be explained by the difference in the type and quality of coating and distribution of the particles in the polymer matrix as well as the testing method. The incorporation of Closite A93 and Al_2_O_3_ into the resin reduced the BFS of the resin. This poor compatibility of silanated Al_2_O_3_ explains the low strength of its composite. In addition, this result is comparable with the results of Abboud et al., [26] who showed that the incorporation of silanated alumina particles into the commercial PMMA reduced its compressive strength. Adding commercial silanated silica had no statistically significant effect on BFS of the resin. This may be due to the hydrolysis of the oxane bonds (Si-O-Si), which was already shown in the tube test. This result is consistent with the result of another study in which adding treated and untreated silica particles to the heat-cure PMMA denture base materials did not improve its transverse bend or impact strength [35]. The BFS of the composite containing 2 w % of the particles, showed a higher strength compared to the ratios of 3 and 5 w %, probably due to the agglomeration of the particles. Acrylic coated glass flakes–PMMA 1 w % composite showed a higher BFS compared with the plain resin however, its impact strength was not significantly higher than plain resin. Similar to the BFS, the impact strength of the resin decreased when a higher ratio of the filler particles was added, which indicates a probable clustering of the particles. The fracture toughness of this composite was not different from the plain resin. Improvement of BFS, while the fracture toughness is unchanged, can be interpreted as a reduced inherent flaw size (K*_I_* = ↑σ√π*a*↓). This means that acrylic coated glass flakes are integrated with the matrix resin.

### 5.3. Mechanical Properties of High-Impact Resin

There was no significant difference between the BFS of the high-impact resin and plain acrylic resin. Yield strength of the high-impact resin is expected to be lower than that of plain acrylic resin due to rubbery particles in its structure [36] and its higher stress corrosion rate [36,37]. The acrylic coated glass flake-high-impact resin composite of 1 w % also showed the same BFS as the tested high-impact resin. The possible explanation for the lack of improvement in BFS of this composite is that the glass flake was introduced only into the matrix of the resin and not in the whole polymer bulk. This forms almost one-third of the polymer bulk and therefore, the weight ratio of the glass flake in the matrix was around 2% to 3%, which was shown in the previous experiment to have the same BFS as the plain resin. Despite statistically insignificant differences, the high-impact resin exhibited 6% higher impact strength compared to the plain acrylic resin, and glass-flake high-impact composite of 1 w % demonstrated an increase of 12% impact strength compared to the high-impact resin itself. If there is a clinically significant difference between the performance of the high-impact resin and plain acrylic resin, it can be concluded that there should be also a clinically significant difference between glass flake added Enigma high base and pure Enigma high base. Thus, a clinical trial study is suggested to investigate the clinical performance of these resins in the oral environment. As expected, the mean fracture toughness of high-impact resin was higher than the plain resin [36]. Although adding acrylic coated glass flakes into the high-impact resin improved its mean fracture toughness further, the differences still were not statistically significant. An unchanged BFS and a slightly increased mean fracture toughness of glass flake filled high-impact resin in comparison with the plain high-impact resin indicates an increase in inherent flaw size of the composite (↑K*_I_* = σ√π*a*↑) probably due to the concentration of the particles in the matrix (2–3 w %) and resulted in increased agglomeration.

It has also been shown by Cazzaniga, Ottobelli [38], that the surface roughness of the resin composites are affected by type and size of the filler particles, which in turn can affect the other mechanical properties such as bending strength and fracture toughness.

As an invitro study, the results should be interpreted within its limitations. Further assessment of surface characteristic of filler particles and their distribution in resin matrix is needed to find out the nature of interaction between different phases of composite resins.

## 6. Conclusions

Silane coupling treatment can modify the surface of inorganic particles making them compatible with acrylic monomer. The success of treatment varies depending on the type of particles and treatment method. Surface modified particles could improve the mechanical properties of PMMA as a denture base resin, provided a uniform dispersion is achieved. Surface modified particles may improve the mechanical properties of high-impact denture base resins; however, the clinical performance of any altered and high-impacted resins will need to be studied to ensure suitability for denture practice. It can be concluded that the silane treatment regime of TiO_2_ particles should not comprise hydrogen peroxide pre-treatment and rinsing after the coupling agent treatment. In the case of ZrO_2_, hydrogen peroxide pre-treatment must be avoided. Although the chemical compatibility of Al_2_O_3_ particles can be improved by applying hydrogen peroxide prior to silanation, further improvement is still required. Silane coupling agent treatment had no effect on the compatibility of the nanoclays. A dual cure polymerisation technique was developed to polymerise MMA containing uniformly dispersed surface modified filler particles. The results revealed that compatibility of the surface modified particles with resin is not the only factor that may affect their performance as reinforcements; the chemical and physical structure of the particles, their dispersion before and after treatment and the chemical characteristics of their coating layer are also influential factors. Adding acrylic coated glass flakes improved the BFS and impact strength of the resin without affecting its fracture toughness, although clustering of the particles still occurs at higher particle to resin ratios. These particles also improved the impact strength of high-impact denture base resin without affecting its BFS and fracture toughness. Further analysis of surface characteristic of coated particles as well as distribution patterns of the filler particles within the resin matrix is recommended. This would help to understand the relation between the particles and their distribution on mechanical behaviour of the resin composites.

## Figures and Tables

**Table 1:** Comparison of biaxial flexural strength of filler added resin and plain resin, MMA. * Statistically significant compared to the control group.

PMMA Composite	BFS (MPa)	SD
Control (cured MMA)	121	12
ac GF, 1%	146 *	16
ac GF, 2%	131	17
ac GF, 3%	106	11
PMMA-Silica, 1%	100	11
Closite A20, 1%	103	28
Closite B, 1%	109	31
A93, 1%	86 *	9
Al_2_O_3_, 1%	65 *	12
TiO_2_, 1%	113	14
Silica, 1%	116	7
Silica, 2%	131	17
Silica, 3%	123	10
Silica, 5%	126	11
ZrO_2_, 10%	107	16

**Table 2:** Effect of adding filler particles on impact strength of resins.

PMMA Composite	IS KJ·m^−2^	SD
Control (cured MMA)	0.92	0.2
ac GF, 1%	1.08	0.32
ac GF, 2%	0.97	0.23
ac GF, 3%	0.84	0.03

**Table 3:** Effect of adding filler particles on fracture toughness of the resin.

PMMA Composite	FT MPa·m^1/2^	SD
Control (cured MMA)	1.86	0.25
ac GF, 1%	1.9	0.22

**Table 4:** BFS of high-impact resin with and without added glass flakes, compared to plain denture resin.

	Plain	HI	HI+acGF1%
BFS MPa	170	153	152
SD	40	16	13

**Table 5:** Impact strength of plain acrylic denture base resin, high-impact denture base resin and its composite with acrylic coated glass flakes.

	PLAIN	HI	HI+GF1%
IM KJ·m^−2^	1.48	1.58	1.77
SD	0.06	0.16	0.47

**Table 6:** Fracture toughness of the plain acrylic denture base resin, high-impact denture base resin and its composite with acrylic coated class flakes. * Statistically significant compared to the plain resin.

	PMMA	HI	HI+GF1%
FT MPa·m^1/2^	2.24	3.02 *	3.43
SD	0.38	0.25	0.78
